# Editorial: Traumatic stress disorders

**DOI:** 10.3389/fnins.2023.1207953

**Published:** 2023-05-17

**Authors:** Hu Zhao, Chunxia Yan

**Affiliations:** ^1^Faculty of Forensic Medicine, Zhongshan School of Medicine, Translational Forensic Medicine Engineering Technology Research Center, Sun Yat-sen University, Guangzhou, China; ^2^Guangdong Province Key Laboratory of Brain Function and Disease, Sun Yat-sen University, Guangzhou, China; ^3^College of Forensic Medicine, Xi'an Jiaotong University Health Science Center, Xian, China

**Keywords:** traumatic stress disorders, pathogenesis, biomarkers, evidence-based treatment, prevalence

Traumatic stress disorders (TSDs) are a group of mental health conditions that result from exposure to traumatic events. Post-traumatic stress disorder (PTSD) is the most well-known and studied TSD. PTSD poses a high burden for individuals and societies. Overall costs for PTSD account for ~43,000 EUR per individual, which is three times higher than costs for non-exposed controls. Of these costs, 59% are caused by mental disorders, 18% specifically by PTSD (Bothe et al., 2020). However, the etiology of TSD, especially PTSD, is currently unclear, and effective treatments are limited.

Translational studies on TSDs involve studying the underlying mechanisms that lead to TSDs, and translating this knowledge to improve clinical outcomes and evaluation for individuals affected by stress. A key focus of this research is identifying new targets for molecular interventions, such as drugs or other therapeutic interventions, to prevent or treat TSDs. Another key area of focus is developing better diagnostic tools or biomarkers to aid in the identification and diagnosis of these disorders. Ultimately, translational studies on TSDs aim to improve the lives of individuals affected by stress-related disorders, by providing more comprehensive and effective prevention and intervention strategies.

The prevalence of PTSD in special population, and risk factors to PTSD susceptibility were explored in three articles of this Research Topic. As a special population, college students received more attention during the COVID-19 pandemic, because they are particularly vulnerable to stress during the ongoing pandemic (Tang et al., 2020). The study of Wang et al. examined the prevalence of PTSD in college students 1 month after the COVID-19 outbreak to identify possible risk factors for PTSD symptoms. More than 1 month after the COVID-19 outbreak, 13.1% of college students developed PTSD symptoms, and they were more likely to experience strained relationships with their family, and drop out of school because of close contact with COVID-19 patients. Neuroticism, psychoticism and an avoidant coping style were associated with increased risk for PTSD symptoms, while an active coping style was protective against PTSD during this pandemic. In addition, previous studies suggested that the DRD2/ANKK1 rs1800497 C>T polymorphism plays a critical role in the risk of PTSD, but published data are inconsistent or even contradictory. Therefore, Niu et al. conducted a meta-analysis on 12 observational studies involving 5,515 subjects to explore the underlying correlation between the rs1800497 C>T polymorphism and PTSD risk. Pooled results revealed an elevated PTSD risk in mutated homozygote TT carriers in the general population (TT vs. CC: OR = 1.73, 95% CI = 1.14–2.62, *P* = 0.01, I2 = 58.9%) and other specific subgroups. Meanwhile, PTSD not only occurs in healthy individuals, but also in patients with cerebrovascular diseases, cancer, and other conditions. The prevalence of PTSD ranged from 3 to 31%, with a weighted proportion of 16.5% in stroke population (Tang et al., 2022). Suffering from PTSD not only decreases stroke patients' quality of life, but also relates to their non-adherence to treatment. Jiang et al. recruited 170 patients with a first-ever stroke during the acute phase. They were randomized into Psycho-therapy group 1 and Control group 1, and were administered with preventive intervention for PTSD or routine health education, respectively. At 2-month follow-up, participants who were diagnosed with post-stroke PTSD were further randomized into Psycho-therapy group 2 and Control group 2, and received supportive therapy or routine health counseling, respectively. At 6-month, they found that the supportive therapy did have a fine effect in ameliorating symptoms for diagnosed PTSD patients, superior to routine health counseling.

The other three articles of this Research Topic are all animal studies. A previous study reported that apoptosis could be one of the most important neuropathological mechanisms for cell loss or hippocampal atrophy induced by predatory stress (Zhao et al., 2007). In the study of Chen et al., they revealed that chronic social defeat stress caused oligodendrogenesis impairment in the medial prefrontal cortex (mPFC) and the lateral habenula in adolescent mice exposed to social defeat stress. Additionally, a major challenge in treating PTSD remains the significant variability in responsiveness to pharmacotherapy. Only 20–30% of patients experience total remission to a specific treatment, while others demonstrate either partial remission or no response. Therefore, Sarkar et al. examined the possibility of employing an “individual behavioral profiling” approach, originally developed to differentiate between “affected” and “exposed-unaffected” individuals in an animal model of PTSD, which will also enable dissociating “responders” or “non-responders” after SSRI (fluoxetine) treatment. The results showed that the ability to respond to fluoxetine treatment may be linked to the ability to modulate excitation-inhibition balance in the hippocampus. They proposed that the “individual behavioral profiling” approach may be employed as an effective translational tool to assess pharmacotherapy treatment efficacy in animal models of stress and trauma-related psychopathology. At the same time, Desnouveaux et al. also developed an ethological model of simulated predator exposure in rats to evaluate the value of electrocorticogram (ECoG) characteristics as a biomarker in predicting stress-induced behavioral phenotypes. They found that decreased chronic 24h frontal Low θ relative power was associated with resilience, increased frontal Low θ relative power was associated with fear memory, and decreased parietal β2 frequency was associated with the avoidant-anhedonic phenotype.

The articles of this Research Topic highlighted the prevalence and risk factors of PTSD in college students and stroke patients, and explored the specific brain damage induced by social psychological stress based on animal model. Meanwhile, the “individual behavioral profiling” approach was developed to predict drug efficacy, and ECoG biomarkers were used to determine stress-related behavior phenotype. Despite progress in the field of translational studies on TSDs, there are still significant shortcomings that need to be addressed. One major challenge is the lack of reliable biomarkers for PTSD and other stress-related disorders, which makes diagnosis and treatment difficult. Another major challenge is the complexity of stress-related disorders, which involve complex interactions between biological, psychological, and social factors. Future research should focus on identifying biomarkers with reliably predictive ability for the onset of TSDs and tracking treatment progress, and developing more integrated approaches to study these complex interactions and more effective treatments.

## Author contributions

HZ wrote the initial draft. HZ and CY critically reviewed the manuscript. All authors contributed to the article and approved the submitted version.
